# Primary carcinoid tumor of medulla spinalis: case report and review of the literature

**DOI:** 10.1186/s40001-014-0071-7

**Published:** 2014-12-19

**Authors:** Xian-feng Zhang, Yan Zhang, Xu Yan, Li Bie

**Affiliations:** Department of Neurosurgery of the First Clinical Hospital, Jilin University, 71 Xinmin St, Changchun, 130021 China; Department of Pathology of the First Clinical Hospital, Jilin University, 71 Xinmin St, Changchun, 130021 China; Changchun Aier Eye Hospital, 71 Xinmin St, Changchun, 130021 China

**Keywords:** Carcinoid tumor, Medulla spinalis, Neuroendocrine tumor, Spinal tumor

## Abstract

**Background:**

Carcinoid tumors are slow growing neuroendocrine tumors which can originate from various sites within the body. A carcinoid tumor originating in the medulla spinalis has not previously been reported in the literature.

**Case report:**

We report a case of a 33-year-old man, presenting with a five-month history of bilateral lower extremity pain, as well as paresthesia, and mild weakness in one lateral lower extremity. A lumbar laminectomy of L3 to L5 and en bloc resection of the tumor was performed. Postoperative histopathology and immunohistochemical analysis of the tumor were consistent with that of a carcinoid tumor. There were no clinical or radiological signs of tumor recurrence or metastasis at the patient’s two year postoperative follow-up.

**Conclusions:**

During the differential diagnosis of medulla spinalis tumors, the possibility of a primary carcinoid tumor originating within the medulla spinalis should be considered. An accurate tumor classification is imperative to ensure that the most effective course of treatment is pursued.

**Electronic supplementary material:**

The online version of this article (doi:10.1186/s40001-014-0071-7) contains supplementary material, which is available to authorized users.

## Background

Carcinoid tumors (CTs) are neuroendocrine neoplasms that generally arise from enterochromaffin cells [1]. These neoplasms are classified as neuroendocrine tumors (NETs) by the World Health Organization [2]. Malignant CTs rarely lead to spinal metastases and typically only occur during the late stages of malignancy [3]. Primary CTs in the spine are extremely rare and have only been described in the cervical, sacral [4,5], and coccygeal [6] spine, or the filum terminale [7,8]. To the best of our knowledge, this is the first reported case of a primary CT of the medulla spinalis.

## Case presentation

### History

A 33-year-old man presented with a five-month history of bilateral radiating pain through the hips, legs and ankles. Additionally, he reported numbness, paresthesia and mild weakness in one lateral lower extremity. A pin-prick sensation test showed bilateral patchy loss of sensation and vibration in the legs and feet. The patient did not report bowel or bladder problems. He did not exhibit hyper-reflexia and was negative for Babinski’s sign.

**Figure 1:**
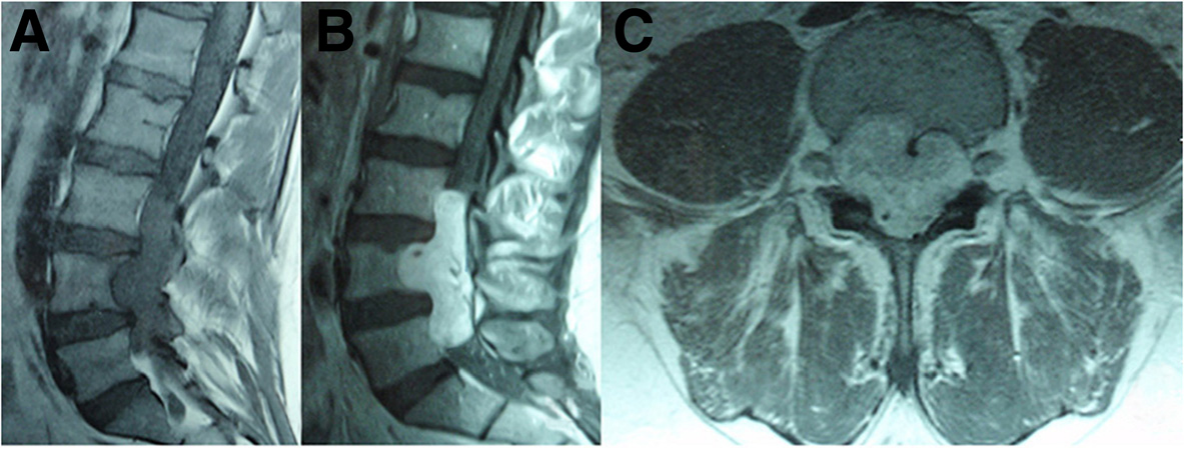
**Pre-operative MR images of the lumbar spine revealed an intradural, extra-medullary, isointense mass behind the vertebral bodies of L3 to L5.** The sagittal T1-weighted image showed the vertebral body of L4 exhibited a compressed deformation **(A)**. The T1-weighted image showed homogenous enhancement with contrast **(B)**. The axial T1-weighted image showed the vertebral body of L4 exhibited a compressed deformation **(C)**.

Magnetic resonance imaging (MRI) revealed a lesion extending from L3 to L5. T1-weighted MRI of the lumbar spine revealed an intradural, extra-medullary, isointense mass behind the vertebral bodies of L3 to L5 that was eccentric to the left and showed homogenous enhancement with contrast. The vertebral body of L4 exhibited a compressed deformation (Figure 1). Extensive examination, including gastroscopy, colonoscopy and computed tomography scans of the abdomen and the thorax, identified no additional masses in the body.

### Operation

A lumbar laminectomy of L3 to L5 was performed. The tumor was soft with a rich blood supply and closely adhered to the medulla spinalis. There were no signs of dural invasion and it maintained a clear separating plane. A total tumor resection was performed.

### Follow-up

**Figure 2:**
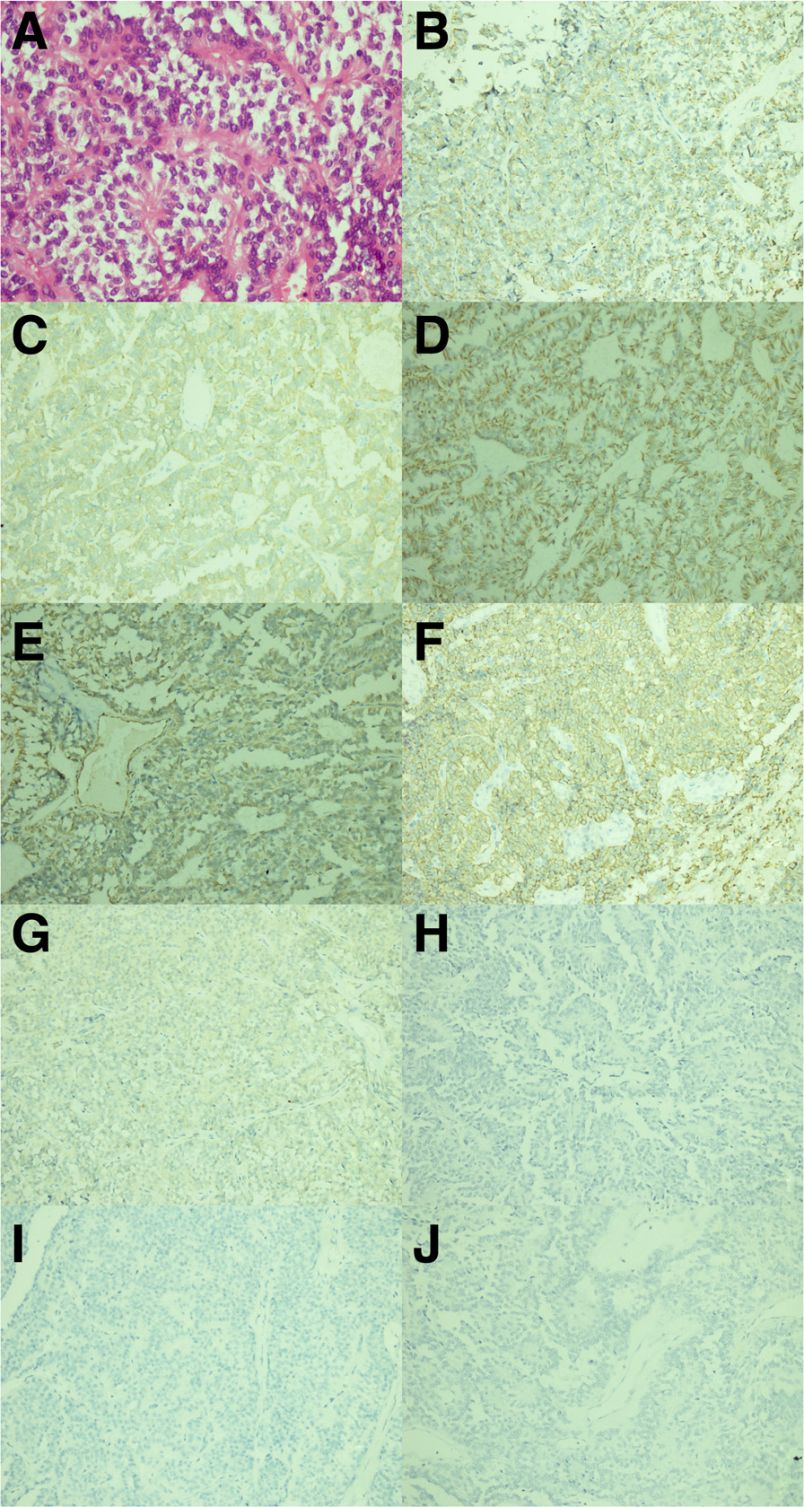
**Microphotographs of specimen histology showed plump round cells with a daisy formation (hematoxylin and eosin staining, magnification × 400). (A)** Microphotographs of specimen histology showed that the tumor cells were positive for chromogranin **(B)**, synaptophysin **(C)**, cytokeratin **(D)**, vimentin **(E)** and CD56 **(F)**, and negative for S-100 **(G)**, GFAP **(H)**, pathogenesis related protein **(I)** and epithelial membrane antigen **(J)**. These findings confirmed the diagnosis of carcinoid tumor (immunohistochemical staining, magnification × 200).

The patient recovered well from the operation. Lower extremity pain resolved immediately following surgery, and lower limb strength began to improve within a few days. The patient reported symptoms of postoperative dysuria, which could be attributed to intraoperative injury to the nerve roots. Dysurea symptoms showed improvement after one week. During the two-year postoperative follow-up examination the patient exhibited no clinical or MRI radiological signs of tumor recurrence or metastasis (Figure 2).

Postoperative histopathology of the tumor confirmed a completely resected solid tumor. The specimen was a fully encapsulated smooth soft-tissue mass. Standard hematoxylin and eosin staining revealed sheet-like proliferation of NET cells in a trabecular pattern and no visible mitoses or necrosis. Additional immunohistochemical staining demonstrated that the tumor was positive for synaptophysin, chromogranin (CgA), cytokeratin and CD56, and negative for S-100 (Figure 2). The Ki-67 labeling index was 2%. These findings are consistent with the known cellular characteristics of a typical CT.

## Discussion

CTs are rare neoplasms that arise from enterochromaffin cells. The estimated incidence of CT is 0.28 to 0.8 per 100,000 individuals [9]. Spinal metastases of CTs are extremely rare and occur in fewer than 2% of cases [10,11].

Reports of diagnosis or therapy of primary or metastatic CTs in the spine are limited, and, as such, it is important to document these individual cases to understand better the optimal treatment for patients.

Patients with spinal CTs typically present with symptoms that are related to the location of the tumor in the spine. The most common symptoms are back and radicular pain, followed by numbness, sphincter dysfunction and motor weakness as a result of spinal cord and/or nerve involvement [10].

Imaging plays an important role in the detection and diagnosis of CT. MRI and computed tomography scans can be performed to reveal spinal cord lesions. Preoperative T1-weighted MRI of the lumbar spine that reveals isointense and contrast-enhanced intradural masses, similar to that described in our case, are more likely to result in a diagnosis of neurinoma or meningioma than CT. In such cases, the pathologic type of the tumor needs to be further clarified by postoperative pathology. Neurinoma and meningioma tumors can be distinguished from CTs based on characteristic histological and immunohistochemical properties [2].

**Figure 3:**
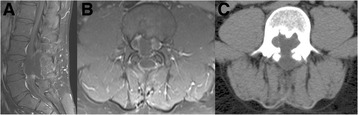
**T1-weighted postoperative MR images of the lumbar spine after contrast application showed no tumor recurrence two years after surgery.** Sagittal T1-weighted postoperative MR images of the lumbar spine **(A)**. Axial T1-weighted postoperative MR images of the lumbar spine **(B, C)**. MR, magnetic resonance.

Total resection of the tumor is the most effective treatment for patients with spinal CTs. In the present case, L4 was compressed and a small portion of the lamina and spinous process were surgically resected. A computed tomography scan revealed that most of the L4 vertebral body, pedicle and facet were intact (Figure 3C). Standing hyperextension, two years after surgery, revealed that no spondylolisthesis was present based on the Meyerding Grading System. The lumbar spine, therefore, exhibited good postoperative stability [see Additional file 1: Figure S1].

Radiotherapy is the best adjuvant option for patients with CTs. It has been reported to increase disease-free and overall survival rates in cases of metastatic CT of the spine. Patients who undergo radiotherapy also achieve clinical palliation and can considerably improve pain and neurological symptoms [12]. Some patients can also benefit from chemotherapy [13]; however, there is limited evidence for chemotherapy and radiotherapy approaches in the management of primary spinal CT.

Further genetic-based studies are likely to elucidate the molecular mechanisms leading to CT and may reveal more effective or individualized therapeutic approaches. Several studies have found that promoter methylation of tumor suppressor genes is associated with the suppression of gene expression and DNA copy number alterations in small intestinal NETs [14,15]. Valentino *et al*. reported that co-targeting the PI3K and RAS pathways effectively inhibits NET cell proliferation and stimulates apoptosis [16]. Swarts *et al*. showed that gene mutations and reduced expression of multiple endocrine neoplasia 1 (MEN1) are associated with poor prognoses in pulmonary carcinoid tumors [17]. Plasma levels of CgA are significantly elevated in CTs and exhibit high sensitivity and specificity as a biomarker for CT diagnosis. CgA can also be used as a tumor development biomarker and for evaluating prognosis during clinical applications [18,19]. Plasma concentrations of tyrosine kinase-2 (Tie-2) have also been proposed as a specific biomarker for NET [20].

Follow-up care relying on multiple imaging approaches is important for the detection of tumor recurrence and metastasis. Recurrence monitoring should include postoperative assessment of 5-hydroxyindoleacetic acid levels in 24-hour urine and plasma CgA levels [21].

## Conclusions

Although rare, primary CTs of the medulla spinalis should be considered in the differential diagnoses of medulla spinalis tumors. Accurate classifications of tumor type will lead to more effective tumor therapy. Surgery is currently the most effective treatment technique for spinal CTs. The use of radiotherapy and chemotherapy for patients should be considered on an individual basis.

## Consent

Written informed consent was obtained from the patient for publication of this case report and accompanying images.

## Additional file

Additional file 1:
**Standing hyperextension two years after surgery revealed that no spondylolisthesis was present based on the Meyerding Grading System.**

## Abbreviations

CTs: carcinoid tumors
NETs: neuroendocrine tumors
MRI: magnetic resonance imaging
Syn: synaptophysin
CgA: chromogranin A
CK: cytokeratin
EMA: epithelial membrane antigen
PR: pathogenesis related protein
MEN1: multiple endocrine neoplasia 1
Tie-2: tyrosine kinase-2

## References

[1] Buchanan KD, Johnston CF, O'Hare MM, Ardill JE, Shaw C, Collins JS, Watson RG, Atkinson AB, Hadden DR, Kennedy TL, Sloan JM (1986). Neuroendocrine tumors. A European view. Am J Med.

[2] Klimstra DS, Modlin IR, Coppola D, Lloyd RV, Suster S (2010). The pathologic classification of neuroendocrine tumors: a review of nomenclature, grading, and staging systems. Pancreas.

[3] Arnold PM, Floyd HE, Anderson KK, Newell KL (2010). Surgical management of carcinoid tumors metastatic to the spine: report of three cases. Clin Neurol Neurosurg.

[4] Dujardin F, Beaussart P, de Muret A, Rosset P, Waynberger E, Mulleman D, de Pinieux G (2009). Primary neuroendocrine tumor of the sacrum: case report and review of the literature. Skeletal Radiol.

[5] Schnee CL, Hurst RW, Curtis MT, Friedman ED (1994). Carcinoid tumor of the sacrum: case report. Neurosurgery.

[6] Krasin E, Nirkin A, Issakov J, Rabau M, Meller I (2001). Carcinoid tumor of the coccyx: case report and review of the literature. Spine (Phila Pa 1976).

[7] Abdulazim A, Citak M, Backhaus M, Stienen MN, Horch C (2010). Primary carcinoid tumor of the filum terminale–a case report. Acta Neurochir (Wien).

[8] Locke J, True LD (2001). Isolated carcinoid tumor of the terminal filum. Case report. J Neurosurg.

[9] Modlin IM, Lye KD, Kidd M (2003). A 5-decade analysis of 13,715 carcinoid tumors. Cancer.

[10] Nathoo N, Mendel E (2011). Spinal carcinoid metastasis: rare but important differential diagnosis of a spinal mass. World Neurosurg.

[11] Tanabe M, Akatsuka K, Umeda S, Shomori K, Taniura S, Okamoto H, Kamitani H, Watanabe T (2008). Metastasis of carcinoid to the arch of the axis in a multiple endocrine neoplasia patient: a case report. Spine J.

[12] Li D, Brennan JW, Buckland M, Parkinson JF (2010). Bronchogenic carcinoid metastasis to the intramedullary spinal cord. J Clin Neurosci.

[13] Lal A, Chen H (2006). Treatment of advanced carcinoid tumors. Curr Opin Oncol.

[14] Edfeldt K, Ahmad T, Akerstrom G, Janson ET, Hellman P, Stalberg P, Bjorklund P, Westin G (2013). TCEB3C a putative tumor suppressor gene of small intestinal neuroendocrine tumors. Endocr Relat Cancer.

[15] Fotouhi O, Fahmideh MA, Kjellman M, Sulaiman L, Hoog A, Zedenius J, Hashemi J, Larsson C (2014). Global hypomethylation and promoter methylation in small intestinal neuroendocrine tumors: an in vivo and in vitro study. Epigenetics.

[16] Valentino JD, Li J, Zaytseva YY, Mustain WC, Elliott VA, Kim JT, Harris JW, Campbell K, Weiss H, Wang C, Song J, Anthony L, Townsend CM, Evers BM (2014). Cotargeting the PI3K and RAS pathways for the treatment of neuroendocrine tumors. Clin Cancer Res.

[17] Swarts DR, Scarpa A, Corbo V, Van Criekinge W, van Engeland M, Gatti G, Henfling ME, Papotti M, Perren A, Ramaekers FC, Speel EJ, Volante M (2013). MEN1 gene mutation and reduced expression are associated with poor prognosis in pulmonary carcinoids. J Clin Endocrinol Metab.

[18] O'Connor DT, Deftos LJ (1986). Secretion of chromogranin A by peptide-producing endocrine neoplasms. N Engl J Med.

[19] Sondenaa K, Sen J, Heinle F, Fjetland L, Gudlaugsson E, Syversen U (2004). Chromogranin A, a marker of the therapeutic success of resection of neuroendocrine liver metastases: preliminary report. World J Surg.

[20] Melen-Mucha G, Niedziela A, Mucha S, Motylewska E, Lawnicka H, Komorowski J, Stepien H (2012). Elevated peripheral blood plasma concentrations of tie-2 and angiopoietin 2 in patients with neuroendocrine tumors. Int J Mol Sci.

[21] Warburton R, Keevil B (1997). Urinary 5-hydroxyindole-acetic acid by high-performance liquid chromatography with electrochemical detection. Ann Clin Biochem.

