# Supplementary figures and images for: Primary carcinoid tumor of medulla spinalis: case report and review of the literature
Source: Eur J Med Res. 2014 Dec 19;19(1):71. doi: 10.1186/s40001-014-0071-7 (PMC4272791; doi:10.1186/s40001-014-0071-7)

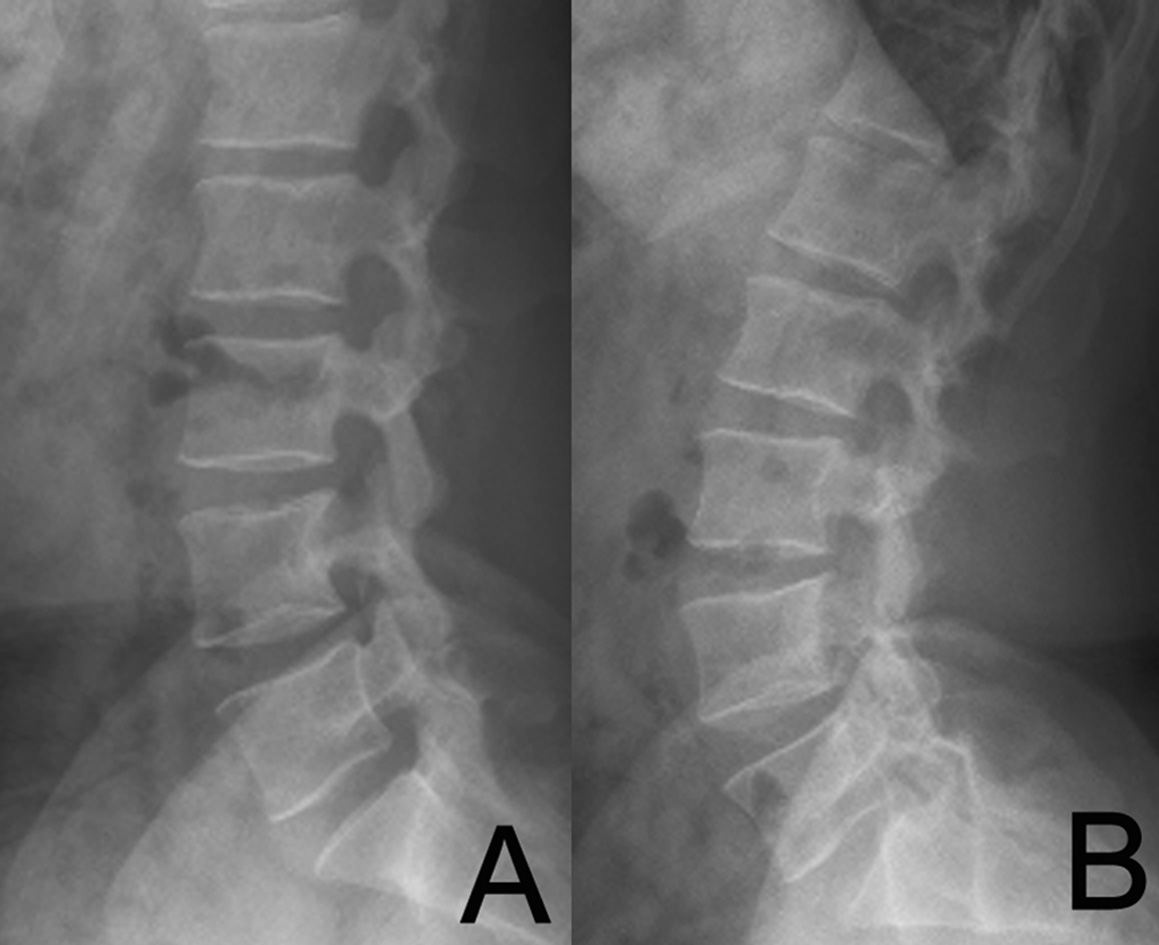

Supplement: Additional file 1: — Standing hyperextension two years after surgery revealed that no spondylolisthesis was present based on the Meyerding Grading System. [file 40001_2014_71_MOESM1_ESM.jpeg]
